# Multidisciplinary management of CDH1 germinal mutation and prophylactic management hereditary lobular breast cancer: A case report

**DOI:** 10.1016/j.ijscr.2019.03.053

**Published:** 2019-04-05

**Authors:** Sara Mirandola, Francesca Pellini, Eleonora Granuzzo, Maya Lorenzi, Beatrice Accordini, Maurizio Ulgelmo, Alessandra Invento, Davide Lombardi, Marina Caldana, Giovanni Paolo Pollini

**Affiliations:** Oncologic Surgery Department, Complex Operative Unit of Breast Surgery – Breast Unit AOUI, Verona, Italy

**Keywords:** CDH1, Lobular breast cancer, Prophylactic bilateral mastectomy, Case report

## Abstract

**Introduction:**

Germline CDH1 mutations, classically associated with hereditary diffuse gastric cancer (HDGC), also imply an increased lifetime risk of developing lobular breast cancer (LBC) in a highly penetrant autosomal dominant manner.

**Presentation of case:**

We report a 44-year-old woman CDH1 mutation carrier with a strong family history of cancer, who previously had prophylactic total gastrectomy. We registered normal findings at the breast and axilla assessment. Mammography, ultrasonography and breast MRI scans were negative for cancer. In our Institute a bilateral prophylactic mastectomy followed by breast reconstruction was performed. Foci of atypical lobular hyperplasia(ALH) and lobular carcinoma in situ (LCIS) were histologically shown.

**Discussion:**

The current consensus guidelines for women with pathogenic CDH1 mutations recommend annual mammography, ultrasound, breast MRI scans and clinical breast examination starting at the age of 35. Due to the well-documented aggressive behavior of this particular type of cancer, bilateral mastectomy and reconstruction would be more beneficial for this kind of high-risk patients.

**Conclusion:**

Conflicting evidences and lacking data about the benefits in terms of overall survival, disease-free survival and the long-term outcomes related to prophylactic bilateral mastectomy for CDH1 mutation carriers restrict the instruction for this type of procedure to selected cases, which should always be managed by a multidisciplinary team.

## Highlights

• Currently, there is no indication to perform prophylactic mastectomy in CDH1 mutation carriers: breast surveillance is recommended.
• To reduce the psychological impact due to bilateral prophylactic mastectomy, that is followed by immediate breast reconstruction.
• A full multidisciplinary team is crucial for the management of patients with CDH1 mutation.

## 1 Introduction

Breast cancer is caused by environmental, lifestyle-related and genetic factors. An important role is played by genetic factors, up to 25% of hereditary cases is due to mutations of highly penetrant genes, such as BRCA1, BRCA2, CDH1, PTEN, TP53 and STK11, each one regulating a specific clinical syndrome [1].

In particular, the lifetime risk of developing hereditary diffuse gastric cancer (HDGC) and lobular breast cancer (LBC) in CDH1 mutation carriers has been estimated to be 80% and 60% respectively [2].

CDH1 gene is located on chromosome 16q22.1 and encodes for E-cadherin, a transmembrane glycoprotein that mediates cellular adhesion. CDH1 mutation is involved in the loss of E-Cadherin expression, which leads to disruption of cell-binding complexes. It results in the loss of cellular cohesion typical of lobular neoplasia [3,4].

In this report we describe our experience in CDH1 mutation carriers management, not well-established in literature yet. Our study outlines how pathogenic CDH1 mutation correlates closely with LBC and that enables an invasive procedure such as bilateral prophylactic mastectomy.

This work has been conducted in line with the SCARE criteria [5].

## 2 Presentation of case

**Fig. 1:**
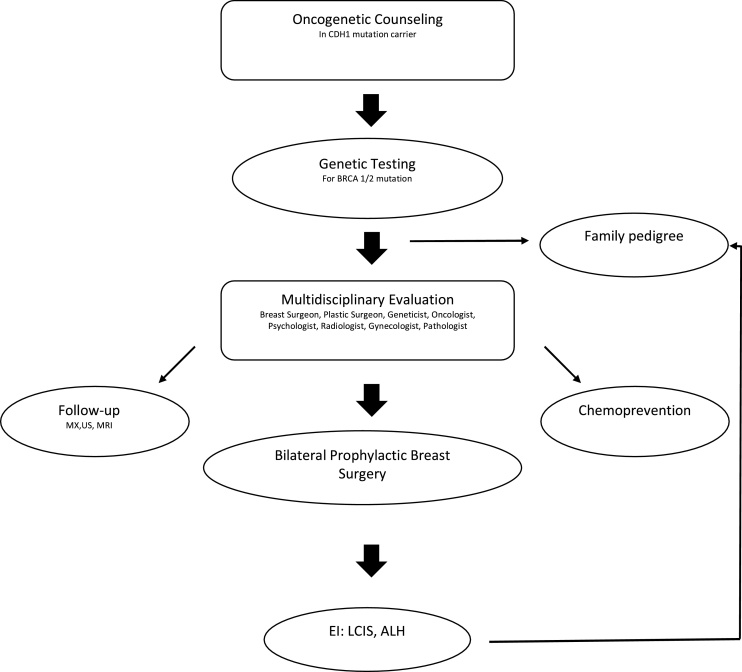
Algorithm for Multidisciplinary Breast Cancer Management in germline mutations (CDH1, BRCA1-2).

A 44-year-old woman with an inherited pathogenic CDH1 mutation visited our department asking for a bilateral prophylactic mastectomy. The patient had no palpable mass, pain or other breast cancer symptoms and she had already undergone prophylactic total gastrectomy. She suffered from epilepsy treated with Tegretol (carbamazepine) due to a cerebral surgery scar after a venous haemorrhage in the parietal lobe (Fig. 1).

Our Institute is specifically committed to breast cancer prevention in patients with hereditary risk. Since January 2016 an Oncogenetic Counseling Service is available to identify people with high risk of developing breast and ovarian cancer and to detect pathogenic genetic mutations.

The patient exhibited medical records which attested genetic test results confirming the patient as a CDH1 mutation carrier. She had also been tested for BRCA mutations with negative results.

The patient’s three-generation family pedigree examination allowed to identify four cases of HDGC and two cases of LBC.

We recorded normal clinical findings at the breast and axilla assessment.

The bilateral prophylactic mastectomy was performed one year after her initial medical exam, during that year we reviewed the updated literature and she was subjected to a preoperative multidisciplinary evaluation including radiological, psychological and plastic surgery examinations.

Mammography, ultrasonography and breast MRI scans were negative for cancer. Neither fine-needle aspiration (FNA) nor biopsy were carried out.

We performed a bilateral nipple-sparing mastectomy with one-stage immediate reconstruction. This technique involves the use of a porcine-derived acellular dermal matrix (ADM) that completely wraps the prosthesis, maintains the pectoral muscle intact and allows implant placement in the subcutaneous tissue. This novel approach can improve aesthetic results and avoid morbidity through the support provided by the biological mesh packed into.

The patient had an uneventful postoperative recovery. Histological evaluation revealed rare foci of atypical lobular hyperplasia and lobular carcinoma in situ.

The resident oncologist’s indication was annual US and breast MRI scans as radiological follow-up.

## 3 Discussion

Atypical lobular hyperplasia (ALH) and LCIS are defined as pre-invasive lesions and non-obligate precursors of breast cancer. Nevertheless, several studies demonstrate that these tumors have a fourfold to fivefold increased risk of developing malignant breast cancer, especially invasive lobular carcinoma [2,3,[6], [7], [8]].

Ma et al. studied molecular aspects and gene-expression of breast atypical hyperplasia, carcinoma in situ and invasive carcinoma. They demonstrated the precursor role of atypical hyperplasia in the development of breast cancer [9].

Even though there are no evidences about an overall survival benefit with bilateral mastectomy in patients with LCIS [10], a Surveillance Epidemiology and End Results (SEER) study analysed 4853 patients diagnosed with LCIS, it revealed a risk of developing invasive breast cancer after LCIS of 7.1% at 10 years [11].

Lobular neoplasia is often diagnosed in premenopausal women, with a mean age at diagnosis of 45 years, it is generally multicentric and bilateral [4].

The aggressive behavior of invasive lobular carcinoma combined with the high penetrance of CDH1 mutations justifies breast Risk-Reducing options.

### 3.1 Radiological and clinical surveillance

The current consensus guidelines for CDH1 mutation carriers recommend annual mammography, ultrasound and breast MRI scans, combined with clinical breast exam starting at the age of 35.^3,20,^

### 3.2 Chemoprevention

Benign lesion (ALH and LCIS) are usually estrogen-receptor-positive and/or progesterone-receptor-positive and human epidermal growth factor receptor 2-negative.

As a result of the National Surgical Adjuvant Breast and Bowel (NSABP) P-1 trial, which demonstrated the effectiveness of Tamoxifen in reducing the onset rate of breast cancer up to 80%, since 1999 FDA approved its use for patients with an elevated risk of future breast cancer [12].

Indeed also Raloxifen, another selective estrogen receptor modulator (SERM), Exemestane and Anastrozole, two aromatase inhibitors, have been approved by FDA for the same reason in postmenopausal women [13].

Preventive therapy for breast cancer with anti-estrogens is effective in high-risk women but their compliance is low, due to the side effects [2].

### 3.3 Surgical approach

Mastectomy is not recommended as a routine procedure in high-risk patients. The lack of data about the risk of developing breast cancer in CDH1 mutation carriers brings risk-reducing surgery currently under discussion.

Invasive lobular cancer is characterized by a tumor density similar or equal to a normal breast tissue density: for this reason conventional imaging, as mammography, is burdened by consistent limitations in detecting this kind of cancer [14].

Moreover, the young onset of malignant breast neoplasia associated with a genetic susceptibility led the American Society of Breast Surgeons (ASCO), the Society of Surgical Oncology (SSO) and the NCCN breast cancer risk-reduction Guidelines to regard bilateral risk-reducing mastectomy as a reasonable option for women with deleterious mutations in BRCA1, BRCA2, CDH1, TP53, PALB2, STK11 or PTEN [8,15].

The prophylactic surgery procedure has been demostrated to confer survival benefits in CDH1 mutation carriers even if it doesn’t completely eliminate breast cancer risk [16].

Preoperative evaluation includes mammography, US and MRI techniques in order to exclude suspicious lesions: in this case conservative mastectomy can be performed.

There are two most commonly followed tecniques: skin sparing mastectomy (SSM), all the breast glandular tissue is removed leaving the skin envelope, and nipple sparing mastectomy (NSM), the nipple-areola complex (NAC) is preserved. The oncological risk of these conservative procedures is due to remaning glandular tissue with the skin flap and/or in NAC: for this reason surgeons should remove all glandular visible tissues and dissect thin skin flap and NAC [17].

Surgery related risks include bleeding, infection, seroma, skin flap necrosis, nipple necrosis, pain. Pathogenic mutation carriers prefer these complications rather than the fear of developing breast cancer [18].

Women that opt for prophylactic surgery can receive immediate reconstruction. This possibility encourages surgical risk-reducing strategies. The implant-based breast reconstruction with acellular dermal matrix (ADM) is the preferred one, because it improves the aesthetic outcomes. Other advantages of pre-pectoral reconstruction consist of avoiding the pectoralis detachment pain, arm weakness and minimizing capsule formation [19].

Postoperative rates of complications such as seroma, red breast syndrome, wound dehiscence, flap necrosis, infection, implant rotation or loss are low: that’s why we can consider this surgical approach useful and safe [20].

The preoperative psychological counseling is a crucial point to investigate the impact of bilateral prophylactic mastectomy and to establish risk factors for postoperative distress. The patient was greatly motivated to approach this prophylactic surgery due to her genetic condition and the fear of leaving her little son orphan. Her psychological status was fundamental in the decision process leading to the bilateral prophylactic mastectomy.

## 4 Conclusion

The recognition of hereditary cancer syndromes is essential in the clinical practice to guide further genetic counseling/testing. Genetic counseling can be provided to discuss detection of one or more alterations and the implications of an identified mutation on a patient’s quality of life.

Early onset of cancer, an increased overall cancer risk, a high probability of second primary tumors conduct to increased medical expenses: therefore it’s necessary to guarantee tailored care for this high-risk population.

Even if many individuals would benefit from the awareness of their hereditary cancer status, this induces also several problems such as coping with their own and their children cancer risk, anxiety, the physical and psychological impact related with prophylactic surgery.

To reduce the psychological impact due to bilateral mastectomy, we used one-step breast reconstruction, named muscle-sparing ADM wrap technique.

This least-invasive technique could improve aesthetic and functional results: reduced hospitalization, rapid return to normal activities and good cosmetic outcomes.

In addition, there are clear economic benefits related to the decreased operating time and days of hospitalization.

To this day there is no uniformly accepted indication to perform prophylactic mastectomy in CDH1 mutation carriers: current consensus guidelines often recommended breast surveillance. In our experience we combined accurate radiological breast monitoring with a dedicated multidisciplinary team to offer the best management of the risk of developing LBC. In some selected cases we consider, instead, that surgical procedure should be performed. We would need more informations about the long term outcomes of women who had elective prophylactic mastectomy and gastrectomy performed.

A full multidisciplinary team is fundamental to focus on all aspects for the management of these patients.

## Conflicts of interest

The authors declare that they have no conflict of interest.

## Funding

This research did not receive any specific grant from funding agencies in the public, commercial, or not-for-profit sectors.

## Ethical approval

No research ethics approval was necessary for this case report.

## Consent

Written informed consent was obtained from the patient for publication of this case report and accompanying images. A copy of the written consent is available for review by the Editor-in-Chief of this journal on request.

## Author contribution

Sara Mirandola: conceptualization, investigation, data curation, writing (original draft), visualization and supervision.

Francesca Pellini: conceptualization, investigation, data curation, writing (original draft), visualization and supervision.

Eleonora Granuzzo: investigation, data curation, writing (review and editing) and visualization.

Maya Lorenzi: investigation, data curation, writing (review and editing) and visualization.

Beatrice Accordini: investigation, data curation, writing (review and editing) and visualization.

Maurizio Ulgelmo: investigation, data curation, writing (review and editing) and visualization.

Alessandra Invento: investigation, data curation, writing (review and editing) and visualization.

Davide Lombardi: investigation, data curation, writing (review and editing) and visualization.

Marina Caldana: investigation, data curation, writing (review and editing) and visualization.

Giovanni Paolo Pollini: investigation, data curation, writing (review and editing) and visualization.

All authors read and approved the final manuscript.

## Registration of research studies

researchregistry4584.

## Guarantor

Dr’s Sara Mirandola and Francesca Pellini are to be considered the co-guarantor’s for this manuscript.

## Provenance and peer review

Not commissioned externally peer reviewed.

## References

[1] Jatoi I. (2018). Risk-reducing options for women with a hereditary breast cancer predisposition. Eur. J. Breast Health.

[2] Crew K.D., Albain K.S., Hershman D.L., Unger J.M., Lo S.S. (2017). How do we increase uptake of tamoxifen and other antiestrogens for breast cancer prevention?. NPJ Breast Cancer.

[3] McAuliffe P., McAuliffe P. (2015). Lobular carcinoma in situ. Breast Dis. Diagnosis Pathol..

[4] Ginter P.S., D’Alfonso T.M. (2017). Current concepts in diagnosis, molecular features, and management of Lobular carcinoma in situ of the breast with a discussion of morphologic variants. Arch. Pathol. Lab. Med..

[5] Agha R.A., Borrelli M.R., Farwana R., Koshy K., Fowler A., Orgill D.P., For the SCARE Group (2018). The SCARE 2018 statement: updating consensus surgical case report (SCARE) guidelines. Int. J. Surg..

[6] Lee J.Y. (2018). Lobular carcinomas *in situ* display intra-lesion genetic heterogeneity and clonal evolution in the progression to invasive lobular carcinoma. Clin. Cancer Res..

[7] Nutter E.L. (2018). Personal history of proliferative breast disease with atypia and risk of multifocal breast cancer. Cancer.

[8] Nakhlis F. (2018). How do we approach benign proliferative lesions?. Curr. Oncol. Rep.

[9] Ma X.-J. (2003). Gene expression profiles of human breast cancer progression. Proc. Natl. Acad. Sci..

[10] Xie Z.M. (2017). Survival outcomes of patients with lobular carcinoma in situ who underwent bilateral mastectomy or partial mastectomy. Eur. J. Cancer.

[11] Chuba P.J. (2005). Bilateral risk for subsequent breast cancer after lobular carcinoma-in-situ: analysis of surveillance, epidemiology, and end results data. J. Clin. Oncol..

[12] Fisher B. (2005). Tamoxifen for the prevention of breast cancer: Current status of the National Surgical Adjuvant Breast and Bowel Project P-1 study. J. Natl. Cancer Inst..

[13] Cuzick J. (2014). Anastrozole for prevention of breast cancer in high-risk postmenopausal women (IBIS-II): an international, double-blind, randomised placebo-controlled trial. Lancet.

[14] Corso G. (2018). Hereditary lobular breast cancer with an emphasis on E-cadherin genetic defect. J. Med. Genet.

[15] Hoskin Tanya L., Hieken Tina J., Degnim Amy C., Jakub James W., Jacobson Steven R., Boughey Judy C., Rochester M. (2016). Use of immediate breast reconstruction and choice for contralateral prophylactic mastectomy. Surgery.

[16] Gullo I. (2018). Phenotypic heterogeneity of hereditary diffuse gastric cancer: report of a family with early-onset disease. Gastrointest. Endosc..

[17] van Verschuer V.M.T., Maijers M.C., van Deurzen C.H.M., Koppert L.B. (2015). Oncological safety of prophylactic breast surgery: skin-sparing and nipple-sparing versus total mastectomy. Gland Surg..

[18] Krontiras Helen, Farmer Meagan, Whatley Julie (2018). Breast Cancer genetics and indications for prophylactic mastectomy. Surg. Clin. North Am..

[19] Vidya R. (2017). Evaluation of the effectiveness of the prepectoral breast reconstruction with Braxon dermal matrix: First multicenter European report on 100 cases. Breast J..

[20] Weber W.P. (2018). Oncoplastic Breast Consortium consensus conference on nipple-sparing mastectomy. Breast Cancer Res. Treat..

